# Diagnostic accuracy of serological diagnosis of hepatitis C and B using dried blood spot samples (DBS): two systematic reviews and meta-analyses

**DOI:** 10.1186/s12879-017-2777-y

**Published:** 2017-11-01

**Authors:** Berit Lange, Jennifer Cohn, Teri Roberts, Johannes Camp, Jeanne Chauffour, Nina Gummadi, Azumi Ishizaki, Anupriya Nagarathnam, Edouard Tuaillon, Philippe van de Perre, Christine Pichler, Philippa Easterbrook, Claudia M. Denkinger

**Affiliations:** 1 Division of Infectious Diseases, Department of Medicine II, Medical Center – University of Freiburg, Faculty of Medicine, University of Freiburg, Germany, Freiburg, Germany; 2 Centre for Chronic Immunodeficiency, Medical Center – University of Freiburg, Faculty of Medicine, University of Freiburg, Germany, Freiburg, Germany; 3 Department of Infectious Diseases, University of Pennsylvania, PA, Philadelphia, USA; 4 FIND, Geneva, Switzerland; 5 Medicines sans Frontières, Access Campaign, Paris, France; 6 School of Medicine, Boston University, Boston, USA; 7 Global Hepatitis Programme, HIV Department, World Health Organization, Geneva, Switzerland; 8 Sargent College, Boston University, MA, Boston, USA; 9 Pathogenesis and Control of Chronic Infections UMR 1058 INSERM/Université Montpellier/Etablissement Français du Sang, INSERM, 34394 Montpellier Cedex 5, France; 10 Centre Hospitalier Universitaire (CHU) de Montpellier, Département de bactériologie-virologie, Montpellier, France; 11 Department of Medicine II, Medical Center – University of Freiburg, Faculty of Medicine, University of Freiburg, Freiburg, Germany

## Abstract

**Background:**

Dried blood spots (DBS) are a convenient tool to enable diagnostic testing for viral diseases due to transport, handling and logistical advantages over conventional venous blood sampling. A better understanding of the performance of serological testing for hepatitis C (HCV) and hepatitis B virus (HBV) from DBS is important to enable more widespread use of this sampling approach in resource limited settings, and to inform the 2017 World Health Organization (WHO) guidance on testing for HBV/HCV.

**Methods:**

We conducted two systematic reviews and meta-analyses on the diagnostic accuracy of HCV antibody (HCV-Ab) and HBV surface antigen (HBsAg) from DBS samples compared to venous blood samples. MEDLINE, EMBASE, Global Health and Cochrane library were searched for studies that assessed diagnostic accuracy with DBS and agreement between DBS and venous sampling. Heterogeneity of results was assessed and where possible a pooled analysis of sensitivity and specificity was performed using a bivariate analysis with maximum likelihood estimate and 95% confidence intervals (95%CI). We conducted a narrative review on the impact of varying storage conditions or limits of detection in subsets of samples. The QUADAS-2 tool was used to assess risk of bias.

**Results:**

For the diagnostic accuracy of HBsAg from DBS compared to venous blood, 19 studies were included in a quantitative meta-analysis, and 23 in a narrative review. Pooled sensitivity and specificity were 98% (95%CI:95%–99%) and 100% (95%CI:99–100%), respectively. For the diagnostic accuracy of HCV-Ab from DBS, 19 studies were included in a pooled quantitative meta-analysis, and 23 studies were included in a narrative review. Pooled estimates of sensitivity and specificity were 98% (CI95%:95–99) and 99% (CI95%:98–100), respectively. Overall quality of studies and heterogeneity were rated as moderate in both systematic reviews.

**Conclusion:**

HCV-Ab and HBsAg testing using DBS compared to venous blood sampling was associated with excellent diagnostic accuracy. However, generalizability is limited as no uniform protocol was applied and most studies did not use fresh samples. Future studies on diagnostic accuracy should include an assessment of impact of environmental conditions common in low resource field settings. Manufacturers also need to formally validate their assays for DBS for use with their commercial assays.

## Background

It is estimated that 71 million people are infected with hepatitis C (HCV) (defined as those with viraemic infection) and 257 million with hepatitis B (HBV) (defined as HBsAg positive) worldwide [1–4]. Both HBV and HCV infection predominantly affect persons in low and middle income countries [2, 5]. Late complications of HBV and HCV infection are cirrhosis and hepatocellular carcinoma. Overall, there were 1.34 million deaths attributable to these complications in 2015 with a trend towards increasing deaths since 1990 [2]. Most patients are not aware of their infection until they have advanced complications of the disease [6] due to lack of access to and affordability of testing. Just as an increase in access to treatment is important [7, 8] early identification is paramount and also cost-effective [9] to avoid disease complications.

HBsAg and HCV-Ab screening is traditionally done by serology using either laboratory based enzyme-immuno-assay (EIA) or a point-of-care rapid diagnostic test (RDT). While several rapid lateral flow tests exist for HBsAg and HCV-Ab detection, their quality is variable or unknown and in particular there is a paucity of good quality HBsAg RDTs available on the market [10, 11]. Four HCV-Ab RDTs are WHO prequalified as of August 2017 but none of the HBsAg RDTs have met the requirements for WHO prequalification [12]. The choice of which format of serological assays to use in a programmatic setting will depend on a variety of factors; most importantly, ease of use and the characteristics of the testing site, such as storage facilities, infrastructure, level of staff skills and cost. Confirmation of active viraemic infection is done using nucleic acid testing (NAT) for HCV RNA and HBV DNA or using serological assays for detection of HCV core antigen.

In order to facilitate more widespread uptake of testing for HBV and HCV, there needs to be greater access to diagnostic assays. The use of dried blood spots (DBS) for transportation using fingerstick (in adults and older children), or heel pricking (in neonates and infants) sampling of capillary blood and subsequent analysis with automated high-throughput laboratory-based EIA represents another affordable alternative to testing using RDTs, particularly in settings with limited infrastructure. Another advantage of DBS use in low resource settings [13] is that capillary blood collection does not require a trained health worker to perform venipuncture and that DBS utilizing capillary blood needs less volume than venepuncture. Furthermore, no centrifuge or even basic laboratory facilities with electrical power are needed to prepare plasma [14]; and since transport and handling do not require high skill or a cold chain, the risk of contamination is reduced [15]. The main disadvantage of DBS is that the existing commercial assays have not been validated or received regulatory approval with this method of sample collection and transport.

Despite this limitation, DBS have been increasingly used in recent years to screen for a number of viral diseases, including HIV and viral hepatitis [13, 16–19]. Some studies have suggested that the use of DBS may increase uptake of hepatitis testing among certain vulnerable risk groups [20–22]. While there have been several systematic reviews on diagnostic performance of RDTs for HBsAg and HCV-Ab [10, 11, 23] and on the use of POC tests in viral hepatitis testing [10, 24, 25], and various validation studies of diagnostic accuracy studies aiming to validate DBS have been performed [26, 27] including a systematic review of HCV RNA detection with DBS [28], to our knowledge there has been no summary of evidence on diagnostic accuracy for HBsAg and HCV-Ab testing using DBS. We have conducted two systematic reviews and meta-analyses: one on the diagnostic accuracy of HCV-Ab and the other on the diagnostic accuracy of HBsAg from DBS samples compared to venous samples in persons identified for hepatitis testing. This review informed the WHO guidelines on testing for chronic HBC and HCV in low and middle income countries [29] and was conducted in conjunction with systematic reviews on the diagnostic accuracy of the detection of HBV DNA and HCV RNA from DBS [30].

## Methods

### Search strategy and selection criteria

PRISMA reporting guidelines were followed [31] and the QUADAS-2 tool was used to estimate quality of studies as a risk of bias tool [32]. We conducted two systematic reviews and meta-analyses on the diagnostic accuracy of HBsAg and HCV antibody detection from DBS compared to venous blood. We searched English language studies using five databases (MEDLINE, Web of Science, EMBASE, Global Health and LILACS) with the following search terms: DBS, Dried blood spot, Dry blood spot, filter paper, Guthrie paper, hepatitis, hepatitis B, hepatitis C, HBsAg, HBV, HCV, HCV RNA, HBV DNA (adapted to databases) on 1.9.2015 and updated it on 22.8.2017.

Abstracts were included for fulltext review if inclusion criteria were fulfilled (namely DBS samples and plasma or serum samples used for detection of HCV-Ab and/or HBsAg) or if exclusion of the abstract could not be performed solely on the basis of the information of the abstract. Eligible studies included comparisons of an index test HCV-Ab and HBsAg using DBS with a reference test HCV-Ab and HBsAg using serum or plasma and following outcomes were analysed: correlation, regression coefficient, specificity, sensitivity and positive/negative predictive values. We included studies regardless of whether the assay used was commercially available or used an in-house technique, and testing on DBS and plasma/serum did not have to use the same assay. There were no date, geographic or population demographic restrictions, and individuals of all age groups were included.

### Screening and data extraction

Two independent reviewers performed title, abstract and full-text review to identify eligible studies. Disagreements were resolved by consent of the reviewers. The references of articles selected for inclusion were also reviewed for additional articles to review. The same data extraction procedure was performed in duplicate for each study and included the following variables: author, publication and study dates, country, percentage of children and adults, age range, gender distribution, type of specimen used for DBS, specimen used as gold standard (plasma or serum), serological assay used, storage conditions and effect of storage conditions. Additional data and clarifications were sought by contacting study authors where necessary.

### Risk of bias and quality assessment

The QUADAS-2 tool categories of study design, index and reference test conduct and reporting of patient flow were adapted for use to assess the risk of bias in included studies. In particular, studies where there was consecutive sampling of patients were rated as being at low risk of bias, and a case control design as at high risk of bias. Studies reporting use of a consistent protocol for index and reference testing for each sample and description of patient flow were rated at low risk of bias) while lack of reporting or inconsistent use of a protocol were considered at high risk of bias.

### Statistical data analysis

Summary estimates of sensitivity and specificity were generated with a bivariate random effects meta-analysis using maximum likelihood estimate and 95% confidence intervals. We calculated positive (Sensitivity/(1-Specificity) and negative (1-Sensitivity/Specificity) likelihood ratios directly from the pooled sensitivity and specificity. Several studies did not have sufficient quantitative data to contribute to both sensitivity and specificity - for example no samples with a negative reference test. In such cases, we performed a univariate random effects meta-analysis of the sensitivity and/or specificity estimates separately to incorporate studies that did not report estimates for both. We then compared univariate analyses with the result of the bivariate analysis, in order to make complete use of all the available data.

Heterogeneity was assessed visually from forest plots and by reporting an estimate of τ^2^ corresponding to the variance of the logit-transformed specificity and sensitivity, which can be interpreted as a measure of between-study variability [33]. Stratified analyses were performed by type of assay used for the index test and by storage conditions. Some studies exposed individual samples or subsets of samples to varying storage conditions or used them to define limits of detection. Since these subsets were not necessarily part of the diagnostic accuracy evaluation we included them in the narrative analysis of the impact of storage conditions on the validity of results. Statistical analysis of the data was performed using STATA 14 (StataCorp. 2015. Stata Statistical Software: Release 14. College Station, TX: StataCorp LP).

## Results

### Included studies

**Fig. 1:**
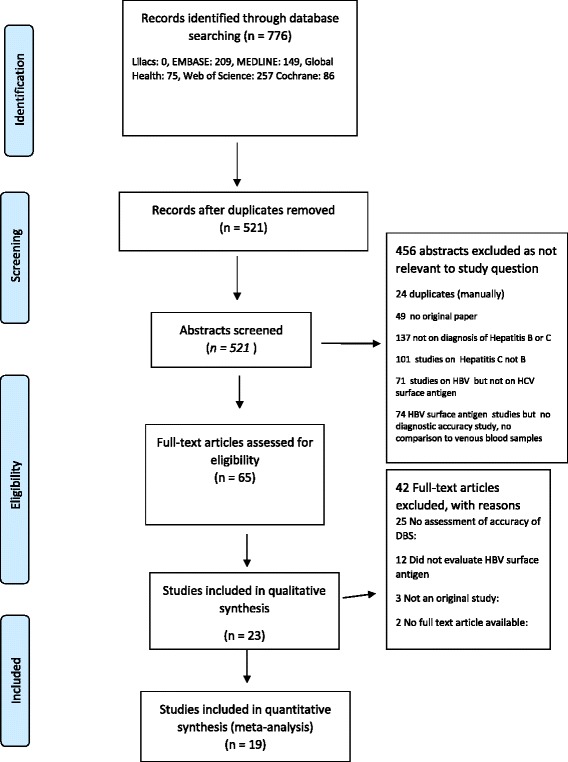
PRISMA flow chart of studies included in the systematic review of detection of hepatitis B Surface antigen from DBS samples compared to venous blood sampling (plasma/serum)

For **HBsAg detection,** our search identified 521 abstracts, of which 65 full text articles were assessed for eligibility, and 23 [34–56] met criteria for inclusion in the review (Fig. 1 & Table 1). Six studies came from Africa [34, 36, 39, 41, 47, 51], nine from Europe [37, 38, 42–44, 50, 53, 55, 56], four from the Americas [35, 45, 46, 54], three from Asia [40, 48, 49]and one from Australia [52]. Four studies did not have sufficient data for sensitivity and specificity analysis [40, 43, 44, 53]. Most studies provided limited information on the characteristics of participants. One study only included pregnant women [35], one only children [51] and one only HIV-infected persons [39]. All studies used the same commercial assays for their reference and their index test, respectively. Five studies from the 1980s used Ausria II (Abbott), whereas newer studies used diverse commercial assays, including Enzygnost (Behring) and ARCHITECT (Abbott) (see also Table 1).

**Table 1:** Characteristics of studies included in the systematic review on HBV surface antigen detection from DBS compared to venous blood sampling

Author, Year, Country	Study design	Study pop, Sample size	Storage conditions	DBS collection method	Serum and plasma antigen test	DBS antigen test	Suggested Cut-off	Specificity	Sensitivity	Correlation/Agreement	Effect of storage conditions
Alidjinou 2013 Congo-Brazzaville	Cross-sectional	Attending hepatology clinic, 32	Temperature: Room temperature (25C) Time: 2 months	Whole blood by venipuncture, 30 μl of plasma onto filter paper	Enzygnost ELISA (Siemens)	Enzygnost ELISA (Siemens)	NR	100	100	Spearman correlation coefficient r of 0.89	No significant change
Boa-Sorte 2014 Brazil	Cross-sectional	Pregnant women, 692	Temperature: Refrigerated Time: Less than 5 days	Unclear amount, venipuncture, Schleicher & Schuell 903© filter paper	IMUNOSCREENHBSAG–SS (Mbiolog Diag.), and Murax HB Sag (Murax BioTech Unlmtd.)	IMUNOSCREENHBSAG–SS (Mbiolog Diag.), and Murax HB Sag (Murax BioTech Unlmtd.)	NR	100	100	NR	NR
Brown, 2012, UK	Cross sectional	Unclear	NR	NR	Abbott Architect I2000	Abbott Architect I2000	NR	100 (unclear N)	98% (unclear N)	NR	NR
Farghaly AM 1990 Egypt	Cross sectional	29 HBsAg pos sera 10 sera positive for anticore only 25 sera negative for any marker of HBV	Temperature: Refrigerated (−20 °C)	5 ml by venepuncture, three drops of blood (50-100 μl) on Whatman No.3	Enzygnost HBsAg	Enzygnost HBsAg	NR	100 (25/25)	100 (29/29)	NR	NR
Farzadegan 1978 Iran	Cross-sectional	10 carriers of HBsAg, 10	NR	Capillary (>0.025 ml) Whatman 4	RIA (Austria II, Abbot), Abbot’s Auscell	RIA (Austria II, Abbot), Abbot’s Auscell	NR	NR	100 (10/10)	NR	Samples stored for 1, 3, 7, 14, and 30 days at 4 °C and 37 °C. Best storage temp °4.
Forbi 2010 Nigeria	Cross-sectional	Broad, 300	Temperature: 4C Time: Overnight	Venipuncture, 25 μl of whole blood, Whatman filter paper	Shantest TM- HBsAg ELISA	Shantest TM- HBsAg ELISA	NR	88.6	78.6	NR	NR
Grune 2015 Germany	Cross-sectional	Inpatients 299	Temperature: −20C, 4C or ambient temperature Time: Up to 14 days	Venipuncture, 100 μl whole blood on paper card	Not specified	Not specified	0.15 IU/ml Derived from different sample	99.8	91.7	NR	NR
Halfon 2012	Cohort	Broad, 200	Temperature: -20C (within 48 h of collection) Time: Not specified	Finger prick, 3 drops whole blood on paper card	BV Cobas Taqman (Roche), Abbott Architect	BV Cobas Taqman (Roche), Abbott Architect	2.8 IU/mL	100	98	NR	NR
Kania 2013 Burkina-Faso	Cross-sectional	Attendees of HIV testing center, 218	Temperature: Ambient temperature Time: Not specified	5 spots whole blood (50 μl) on Whatman 903	Determine, SD Bioline, ETI-MAK-4 HBsAg EIA (DiaSorin S.p.A.), Architect HBsAg QT (Abbott)	Determine, SD Bioline, ETI-MAK-4 HBsAg EIA (DiaSorin S.p.A.), Architect HBsAg QT (Abbott)	Optical density: MAPC (mean absorbance of positive control)/2 + 0.3 standard deviations = 0.825.	100	96	Kappa: 0.98	NR
Khan 1996 Pakistan	Cross sectional	Broad, 90	Temperature: 2–8 °C Time: overnight	Venepuncture, NR	Enzygnost, HBsAg monoclonal II by Behring	Enzygnost, HBsAg monoclonal II by Behring	Cut off value (according to figures: 0.116 mean absorbance value)	100 (86/86)	100 (4/4)	NR	NR
Lukacs Germany 2005	Unclear	70 random samples from newborns (as negative controls), 8 hepatitis b patients	Temperature: NR Time: NR	Venipuncture, NR	NR	Luminex 100 reader, SREPE	Cut-off median fluorescence intensity 233 (mean + 2 Standard deviations for healthy controls)	NR	100% (8/8)	NR	NR
Lee 2011 Malaysia	Cross-sectional	Patients at a tertiary hospital, 150	Temperature: -20C Time: NR	Venipuncture, 50 μl whole blood on filter paper	Abbott, Pearson Test	Abbott, Pearson Test	Cut-off point of 1.72 Relative light units	98	97	Pearson value, *r* = 0.43	NR
Mayer 2012 US	Cross-sectional		Temprature: ambient temperatures Time: Not specified	50 μl on Whatman (8 mm)	ADVIA Centaur 5634 W	ADVIA Centaur 5634 W		100 19/19	100 44/44	*r* = 0.99	Samples stored at ambient temperature and assayed for month four months every week with HBsAg concentration stable
Mendy 2005 The Gambia	Cohort	Broad, 166	Temperature: 30-33C (humid conditions) Time: Up to 4 weeks	Three to five drops of blood (20 μl) on filter paper	Determine HBsAg (Abbott Laboratories)	Determine HBsAg (Abbott Laboratories)		100	96	NR	NR
Mohamed 2013 France	Cohort	Broad, 200	Temperature: Room temperature Time: 1, 3, 7 and 14 days	Venipuncture 50 μl on 12 mm discs (Whatman)	Chemiluminescent microparticle immunoassay (Abbott)	Chemiluminescent microparticle immunoassay (Abbott)	0.30 +/−0.81 IU/mL.	100	98	*r* = 0.85	No significant change
Mossner Denmark 2016	Cohort	404 Prospective patients from hepatitis clinic and blood donors	Temperature: Room temperature Time 1–5 days	Finger prick, 75 μ on Whatman filter paper	Architect HBs Ab and Architect HBc Ab assays using the Architect system (Abbott Diagnostics, Delkenheim, Germany)	Architect HBs Ab and Architect HBc Ab assays using the Architect system (Abbott Diagnostics, Delkenheim, Germany)	The DBS negative patient had serum quantitative level of HbsAg 175 IU/mL.	100% 318/318	98% 84/85	NR	Variation of 24 h to up to 7 d found no difference in stability of samples
Nielsen, 1980, Germany	Cross sectional	110 patients of both sexes older than 6 years attending outpatient department of the General Hospital Barmenda, Cameroon	Temperature	Venipuncture, 50 μl on 16 mm diameter filter paper, Schleicher-Schüll	RIA (Ausria II-test)	RIA (Ausria II-test)	NR	NR	100 11/11	NR	NR
Parkinson, 1996, Alaska	Cross sectional	Alaska Native carriers of HBsAg, volunteer and pregnant females, 10	Temperature and Time NR	Venipuncture, 100 μl applied to 1,5 cm diameter filter paper (No 903, Schleicher &Schuell)	RIA (Ausria II-test)	RIA (Ausria II-test)	Limit of detection approximately 1.25 ng/ml in blood spots	NR	100 (10/10)	NR	Detectable after 8 weeks of storage at ambient temperatures
Ross 2013 Germany	Cross-sectional	Broad, 299	Time and temperature not specified	Venipuncture, 100 μl applied to filter paper (Whatman)	Abbott ARCHITECT HBsAg	Abbott ARCHITECT HBsAg	15 IU/ml (HBsAg)	100	99	*r* = 0.86, good agreement in Bland-Altmann plot	NR
Villa Italy 1981	Cross-sectional	Patients attending liver clinic, 24	Temperature: -20C 4C and room temperature. Time: 1,7, 15, 30, 60, and 180 days	Capillary blood on Whatman filter paper	Ausria II, Ausab, Corab, e-anti e RIA kit (Abbott)	Ausria II, Ausab, Corab, e-anti e RIA kit (Abbott)	Unknown	100	100	NR	Temperature: Storage at room temperature resulted in no significant change compared to samples stored at 4C or -20C. Time: Storage greater than 15 days negatively affected sensitivity.
Villar Brazil 2011	Cross sectional	Patients attending HCVlinic, 133	Temperature:-20C, 4–8C, 22–25C, and 22–25C. Time:1, 7, 14, 21, 42, 63, 112, and 183 days.	either a finger prick or from 70 μl of a whole blood sample onto the 903 Specimen Collection Paper, Whatman Protein Saver Card	ETI-MAK-4 (Diasorin)	ETI-MAK-4 (Diasorin)	Absorbance value 0.115	97	98	NR	Accuracy of DBS samples was stable over 63 days at all temperatures evaluated but after 63 days, accuracy diminished when stored at 22–25C
Zhuang Australia 1982	Cross-sectional	90 sera selected from serum collection at Fairfield Hospital, Australia	Temperature: incubated at room temperature Time: 1 night	200 μl on filter-paper (glass-fibre paper, cut into 3x3cm squares)	Ausria II-125	RIA	NR	NR	100 90/90	NR	Storage at 4 °C, ambient temperatures and 37 °C up to 1 month did not lower sensitivity
Zoulek Sao Tome and Principe 1983	Cross sectional	86 children (48 boys, 38 girls, mean age: 8,3 + − 8,5)	Temperature: stored at 4 °C for 2 weeks, −20 °C for weeks Time: 2 weeks, then unspecified	Venepuncture 2–4 drops (50-100 μl) on 1 cm filter paper discs	RIA Abbot (unspecified)	RIA Abbot (unspecified)	Lowest concentration analysed 0,1 mg/l	NR	100 24/24	NR	NR

**Fig. 2:**
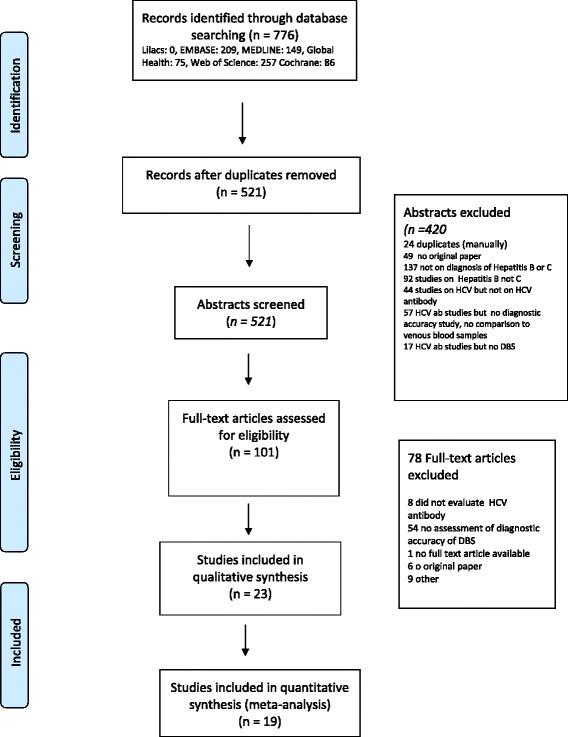
PRISMA flow chart of studies included in the systematic review on detection of hepatitis C antibody detection from DBS samples compared to venous blood sampling (plasma/serum)

For **HCV-Ab detection,** our search identified 521 abstracts, of which 101 full text articles were assessed for eligibility, 23 studies met the criteria for inclusion in the review [26, 27, 37, 39, 40, 43, 55–71]. Nineteen had sufficient data for inclusion in the quantitative meta-analysis (Fig. 2 & Table 2). Four studies did not have sufficient data for sensitivity and specificity calculations [60, 61, 67] or described a Receiver Operating Characteristic (ROC) curve only [40], and contacting the authors did not yield further information. Two of the 19 studies provided sufficient data to calculate sensitivity but did not have any negative samples to assess specificity [37, 56] (Fig. 2 & Table 2). Of the 23 studies included in the review, most originated from Europe, North America and Australia (16 in total). Four studies were from South America [26, 59, 69, 70] and three from South-East and Central Asia (India [62], Mongolia [60] and Malaysia [40]). All included studies had been published between 1997 to 2017. Most studies used 50 μl to 100 μl of whole blood on filter paper to test for HCV-Ab. Six studies reported using capillary blood samples [26, 55, 57, 58, 63, 66], while others either did not report on this or used venous blood. One study included children [68], however, age ranges or gender for patients were not always reported. Various assays for detection of HCV-Ab in serum and DBS were used (see also Table 2).

**Table 2:** Characteristics of studies included in the systematic review on HCV antibody detection from DBS compared to venous blood sampling

Author, Country	Study design	Study pop, Sample size	Storage conditions	DBS collection method	Serum and plasma antibody test	DBS antibody test	Suggested Cut-off	Specificity	Sensitivity	Correlation/Agreement	Effect of storage conditions
Brandao Brazil 2013	Cross sectional	386 persons, 40 anti-HCV pos, 346 blood donoers HCV non-reactive,	DBS samples air dried at room temperature for 4 h, stored at −20 °C. Temperature: −20 °C Time: Not specified	capillary blood by finger prick 75 μl onto Whatman filter paper	MonolisaTM HCV AgAb ULTRA, Bio-Rad (Marnes-la-Coquette, France), and Murex HCV AgAb, Abbott (Kyalami, Republic of South Africa).	MonolisaTM HCV AgAb ULTRA, Bio-Rad (Marnes-la-Coquette, France), and Murex HCV AgAb, Abbott (Kyalami, Republic of South Africa).	ROC cutoff: 0.287 nm for Monolisa assay ROC cut-off for Murex assay 0.238 nm Derived from the same sample	99.7 (98.4–99.9) 95.9 (93.3–97.8)	97.5 (86.8–99.9) 97.5 (86.8–99.9)	PPV and NPV calculated Kappa = 0.99 (with ROC cut-off),	stability up to 60 days of storage at room temperature, but less variation at −20 °C
Croom Australia 2006	Cross sectional	103 samples from high risk groups, negative samples from 94 indivdiuals tested at Haematology Lab	Air dried at room temperature, storage at −20 °C, plasma at −20 °C, time of storage 1 week −11 months Temperature: −20 °C Time: 1 week −11 months	Venipuncture, 80 μl of each whole blood sample spotted onto Schleicher and Schuell cards (Grade 903)	Monolisa EIA, confirmation test: Murex anti HCV (version 4.0), EIA	Monolisa EIA, confirmation test: Murex anti HCV (version 4.0), EIA	NR	100% (96–100) 108/108	100% (94–100) 75/75	NR	NR
Chevaliez France 2014	Unclear	529 patients, 183 HCV seronegative, 346 seropositive	NR	NR	EIA HCV assay	EIA HCV assay	0.2	98.9 (96.1–99.7)	99.1 (97.4–99.7)	*R* = 0.56	NR
Dokubo US 2014	Cross sectional within a prospective cohort study of those HCV positive being followed	148 participants in a prospective study of HCV	DBS air-dried for 2 h, then sent to another insitute, then stored at −70 °C	Fingerstick on Whatman 903 cards 0.5 ml blood	Standard diagnostics HCV TMA (Norvatis®) ELISA v3.0(Ortho®).	Standard diagnostics HCV TMA (Norvatis®) ELISA v3.0(Ortho®).		100% (71/71)	70% (54/77)	Kappa 0.69	NR
Flores 2016 Brazil	Cross sectional	Participants recruited from ambulatory and general hospital, known HCV/HIV serological status	DBS airdried 4 h, then frozen at -20C	Venipuncture, Whatman filter paper, 3–5 drops (~75 μl)	HCV Murex AB, Diasorin	HCV Murex AB, Diasorin	NR	100% (99/99)	94% (213/230)	Spearman correlation *r* = 0.520	NR
Gruner 2015 Germany	Cross-sectional	Inpatients, 299	Temperature: -20C, 4C or ambient temperature Time: Up to 14 days	Venipuncture, 100 μl whole blood on paper card	Not specified	Not specified	Derived from different sample	NR	99% 339/343	NR	NR
Kania Burkina-Faso 2013	Cross sectional	218 HIV screening participants, 5 anti-HCV pos, 213 anti-HCV neg	NR	Venipuncture, 5 spots whole blood (50 μl) on Whatman 903	Monolisa HCV-Ab-Ag ULTRA assay (Bio-Rad), further assessment with Inno-Lia HCV Score assay (Innogenetics)	Monolisa HCV-Ab-Ag ULTRA assay (Bio-Rad), further assessment with Inno-Lia HCV Score assay (Innogenetics)	0.439	100% (97,8–100%)	100% (46,3–100%)	kappa: 1,00 (0,93–1,00)	NR
Larrat France 2012	Cross sectional	One hundred thirteen HCV-positive cases consecutively recruited 17 HIV/HCV co-infected patients (15%)	DSB dried 24 h at room temperature	Finger prick blood on Whatman card	Monolisa® HCV-Ag-Ab-ULTRA, Bio-Rad)	Oraquick HCV CEIA Biorad	0.1 0.2 cEIA	100 (95.8–100) 88/88 100 (95.8–100) 88/88	97.4(92.5–99.1) 110/113 98.2 (93.8–99.5) 111/113	ROC AUC OMT cEIA Biorad: 0.99 ROC AUC FSB cEIA Biorad: 0.918	At 3 days room temperature 3/3 HCV negative samples NR, ODs lower in HIV co-infected patients
Lee Malaysia 2011	Cross sectional	150 paired samples	Left to dry overnight at room temperature, then stored −20 °C	Venipuncture, 50 μl whole blood on filter paper	Abbott	Abbott	ROC cut off 0.10 RLU	100%	97.3%	ROC curve AUC: 0.99 *R* = 0.631	NR
Lukacs Germany 2005	Unclear	7 samples from known HCV patients	NR	NR	NR	Luminex	NR	NR	100% 7/7	NR	NR
McCarron UK 1999	Case control	NR	NR	NR	NR	NR	0.99 1.99	87.5% 100%	100% 97.2%	NR	NR
Marques Brazil 2012	Cross sectional	21 and 24 HCV reactive patients, 234 individual and 132 HCV negative	serum stored at −20 °C	Venipuncture, 75 μl whole blood on Whatman paper	Two methods: HCV-Ab Radim, Pomezzia, Italy and ETI-AB-HCVK-4 DiaSorin, Vercelli, Italy	Two methods: HCV-Ab Radim, Pomezzia, Italy and ETI-AB-HCVK-4 DiaSorin, Vercelli, Italy	Radim: manu-facturer’s cut off ROC curve for DiaSorin EIA using the same sample population	99.5% (98–99.9) 98.9% (96.80–99.55)	97.5% (86.84–99.94) 88.9% (75.95–96.29)	NR	2–8 degrees C, 20–25 degrees C, and −20 degrees were evaluated, −20 resulted in lowest variation Methods of cut off determination: the receiver operating characteristic curve(AUROC)
Marques Brazil 2016	Recruited at Viral Hepatitis Lab	99 (59 anti HCV/HCV RNA pos, 40 neg samples)	NR	Venipuncture, 3–5 drops on Whatman filter paper	HCV Ab Radim, Pomezia, Italy	HCV Ab Radim, Pomezia, Italy		100% 40/40	94.9% 56/59		
Mossner Denmark 2016	Cohort	404 Prospective patients from hepatitis clinic and blood donors	Temperature: Room temperature Time 1–5 days	Finger prick, 75 μ on Whatman filter paper	Architect HCV Ab, using the Architect system (Abbott Diagnostics, Delkenheim, Germany)	Architect HCV Ab, using the Architect system (Abbott Diagnostics, Delkenheim, Germany)	NR	100% 288/288	97% 112/116	NR	Variation of 24 h to up to 7 d found no difference in stability of samples
Nandagopal India 2014	Unclear	Murex	60 samples	Venipuncture,50 μl of whole blood 903 Whatman card	NR	NR	NR	100 (29/29)	100 (31/31)	Pearson correlation coefficient 0.98	NR
O Brien US 2001	Multicenter prospective trial (one-arm only)	1286 subjects enrolled in multi-centre study,	Air dry for 30 min, sent in FedEx envelope	Self collected capillary blood with at home kit	NR	HCV Check, Home Access Corp. self use DBS home kit	NR Several inconclusive and indeterminate results not included in diagnostic accuracy calculations	100% 686/686	99.5% 402/404	NR	NR
Parker UK 1997	Case control design	80 anti HCV positive samples, 52 negative 569 dB sample fields from South African neonates	Air dry at room temperature before storage at 4 °C	NR, Dried blood field samples	In house IgG ELISA, immunoblot RIBA 3.0	In house IgG ELISA, immunoblot RIBA 3.0	T/N 5.0 T/N10.0	541/569 95.1%	78/80 98% 69/80 86.2%	NR	
Ross Germany 2013	Unclear	339 samples	Dried overnight at room temperature	Venipuncture 100 μl of whole blood applied to Whatman 903 filter paper	ARCHITECT system (Abbott Diagnostics, Delkenheim, Germany).	ARCHITECT system (Abbott Diagnostics, Delkenheim, Germany).	NR	100% (97.7–100) 160/160	97.8% (96–100) 175/179	NR	NR
Soulier France 2017	Cross-sectional	511 patients recruited, with known serostatus for HCV	Temperature:-80 Time: NR	Venipuncture, 50 μl on Whatman filter paper	EIA; aHCV Vitros ECi; Diagnostics, Raritan, New Jersey).	EIA; aHCV Vitros ECi; Ortho-Clinical Diagnostics, Raritan, New Jersey).	NR	312/315 98%	183/186 99%	NR	25 dB samples stored at ambient temperatures (24 °C) for a mean duration (±SD) of 19 ± 1 months Sensitivity for genotype detection in DBS 84.5%
Sheperd UK 2013	Cross sectional	19 recently infected 300 chronic carrier 82 resolved infection	DBS stored at 4 °C until use	NR, 50 μl on 903 Whatman Protein Saver cards	ORTHO HCV 3.0 ELISA Test System with Enhanced SAVekit (Ortho Clinical Diagnostics) was used to detect anti-HCV in DBS	NR	Avidity cut-off AI < 30	NR	NR	NR	NR
Tejada-Strop US 2015	Case control	103 patients with known HCV result, 33 adult patients with chronic HepC with stored samples	-20 °C until 5 years later	NR, 75 μl of whole blood on 12 mm DBS	Two immunoassays, the VITROS anti-HCV IgG chemi-luminescence assay (CIA) and the HCV 3.0 enzyme immunoassay(EIA), both from Ortho Clinical Diagnostics (Rochester, NY),	Two immunoassays, the VITROS anti-HCV IgG chemi-luminescence assay (CIA) and the HCV 3.0 enzyme immunoassay(EIA), both from Ortho Clinical Diagnostics (Rochester, NY),	3.26 CIA 1.5 EIA Derived in the same sample	Not calculated	CIA 48/52 92% EIA 90% 47/52 For stored samples CIA: 100% (33/33) EIA: 32/33 97%	100% (CIA and EIA)	NR
Tuaillon France 2010	Case control	100 anti HCV pos serum samples and 100 anti HCV neg samples	18 h dried at room temperature, stored at −20°c for 1–8 weeks	NR, 50 μl of whole blood on Whatman 12 mm paper discs	Ortho HCV 3.0 ELISA, immunoblot assay INNO-LIA HCV Score as confirmatory test	Ortho HCV 3.0 ELISA, immunoblot assay INNO-LIA HCV Score as confirmatory test	Threshold value 0.380	98% (97–100)	99% 97–99)	NR	Stability of anti HCV and HCV RNA investigated by varying room temperature exposure 2–12 days until freezing, after 6 days at room temperature ODs > than cut off values
Waterboer Mongolia 2011	Cross sectional	1022 sexually active women from cross sectional study (response rate 69%)	Room temperature up to 8 h, then −20 °C up to 1 month (serum + DBS)	Venipuncture, Whole blood applied to 5 spots on DBS filter paper cards (Whatman 903)	In house, the HCV (strain H77, subtype 1a) Core and NS3 proteins	In house, the HCV (strain H77, subtype 1a) Core and NS3 proteins	Sera 1492 (Core) 371 (NS3) DBS 967 (Core) 310 (NS3)	Not calculable from the data	Not calculable from the data	98% Agreement (kappa 0.94) for Core 96.1% Agreement (kappa 0.90) for NS3	NR

### Assessment of study quality and risk of bias (Tables 3 & 4)

Ten studies investigating **HBsAg** did not use a random or consecutive sampling method [41–48, 50, 55, 56]. Only one study reported on blinding of laboratory personnel to results of the reference test while performing diagnostic tests [35]. Overall, the study quality was rated as moderate. For **HCV-Ab detection,** eight of the included studies used case-control designs [27, 43, 64, 66–69, 71] and only two reported consecutive sampling [39, 40]. Only three studies reported blinding of laboratory personnel to results of the reference test [27, 57, 66]. However, most of the other studies (as with HBsAg detection) used and reported a clear and consistent protocol for both reference and index test, so this was not judged as a major cause of bias.

**Table 3:** Risk of bias in studies included in the systematic review on detection of HB surface antigen

Author	Patient selection	Bias	Index test	Bias	Reference standard	Bias	Flow and timing	Bias
	Was a case control design avoided? Consecutive or random sample of patients? Inappropriate exclusions?		Blinded to reference standard Could the conduct or interpretation of the index test have introduced bias?		Blinded to index? Could the reference standard have introduced bias?		There is an appropriate interval between the index test and reference standard? All patients receive the same reference standard and are included in the analysis?	
Alidjinou	NR, but no case control design	UR	Not blinded, interpretation unbiased	LR	Not blinded, interpretation unbiased	LR	NR	UR
Boa-Sorte	No case control design, consecutive recruitment	LR	blinded	LR	blinded	LR	Same reference standard, all patients included in analysis	LR
Brown	Not reported	UR	Not reported	UR	Not reported	UR	Not reported	UR
Farzadegan	Only cases, no consecutive sampling	HR	Not blinded, interpretation unbiased	LR	Not blinded, interpretation unbiased	LR	NR	UR
Farghaly	Case control design	HR	Not blinded, interpretation unbiased	LR	Not blinded, interpretation unbiased	LR	NR	UR
Forbi	No case control design	LR	Not blinded, interpretation unbiased	UR	Not blinded, interpretation unbiased	UR	Sampling not reported, same reference standard	UR
Gruner	NR	UR	Not blinded, NR	UR	NR	UR	NR	UR
Halfon	NR, probably case control design	UR	Not blinded, NR	UR	Not blinded, NR	UR	NR	UR
Kania	Consecutive recruitment	LR	Not blinded, interpretation unbiased	UR	Not blinded, interpretation unbiased	UR	Sampling reported	LR
Khan	No case control design	LR	Not reported, unclear whether blinded	UR	Not reported, unclear whether blinded	UR	Sampling not reported	UR
Lee	Consecutive recruitment	LR	Not blinded, interpretation unbiased	LR	Not blinded, interpretation unbiased	LR	Sampling reported, same reference standard	LR
Lukacs	Case control design	HR	Not blinded, interpretation unbiased	LR	Not blinded, interpretation unbiased	LR	Sampling not reported, same reference standard	UR
Mayer	NR, probably case control	UR	Not reported, interpretation unbiased	LR	Not reported, interpretation unbiased	LR	Sampling not reported	UR
Mendy	Case control design	HR	Not blinded, interpretation unbiased	LR	Not blinded, interpretation unbiased	LR	Sampling reported	LR
Mohamed	Case control design	HR	Not blinded, interpretation unbiased	LR	Not blinded, interpretation unbiased	LR	NR	UR
Mossner	Sampling from high-risk and low risk groups	HR	Not blinded, interpretation unbiased	UR	Not blinded, interpretation unbiased	UR	Sampling reported, same reference standard, all patients included in analysis	LR
Nielsen	Only cases	HR	Not reported	UR	Not reported	UR	Sampling not reported	UR
Parkinson	Only cases	HR	Not blinded, interpretation unbiased	LR	Not blinded, interpretation unbiased	LR	Sampling not reported, same reference standard	UR
Ross	Sampling not reported, probable case control design	HR	Not blinded, interpretation unbiased	LR	Not blinded, interpretation unbiased	LR	Flow reported	LR
Villa	Case control design	HR	NR	UR	NR	UR	NR	UR
Villar	Case control design	HR	Bias possible, as selective samples by OD values	HR	Not blinded, interpretation unbiased	LR	Sampling reported	LR
Zhuang	No case control design	LR	Not blinded, interpretation unbiased	LR	Not blinded, interpretation unbiased	LR	Sampling not reported	UR
Zoulek	Unclear, but no case control design, probably random or successive	LR	Not blinded, interpretation unbiased	LR	Not blinded, interpretation unbiased	LR	Sampling not reported, same reference standard	LR

Abbreviations: LR: low risk, HR: high risk, UR: unknown risk, NR: not reported; shaded: low risk of bias

**Table 4:** Risk of bias table for HCV antibody

	Patient selection	Bias	Index test	Bias	Reference standard	Bias	Flow	Bias
	Was a case control design avoided? Consecutive or random sample of patients? Inappropriate exclusions?		Blinded to reference standard Could the conduct or interpretation of the index test have introduced bias?		Blinded to index? Could the reference standard have introduced bias?		There is an appropriate interval between the index test and reference standard? All patients receive the same reference standard? All patients recruited into the study are included in the analysis?	
Brandao	No case control design, consecutive sample, no exclusions	LR	Not blinded, interpretation unbiased	LR	Not blinded, interpretation unbiased	LR	Sampling reported, same reference standard	LR
Croom	Sampling from high-risk and low risk groups	UR	Not blinded, interpretation unbiased	LR	Not blinded, interpretation unbiased	LR	All patients included, same reference standard	LR
Chevaliez	NR	UR	NR	UR	NR	UR	NR	UR
Dokubo	No case control, concurrent sampling from a prospective cohort	LR	Not blinded, interpretation unbiased	LR	Not blinded, interpretation unbiased	LR	Sampling reported, same reference standard, all patients recruited included in analysis	LR
Flores	Case control design	HR	Not blinded, interpretation unbiased	LR	Not blinded, interpretation unbiased	LR	Sampling reported, same reference standard, all patients recruited included in analysis	LR
Gruner	NR	UR	Not blinded, NR	UR	NR	UR	NR	UR
Kania	Consecutive recruitment	LR	Not blinded, interpretation unbiased	LR	Not blinded, interpretation unbiased	LR	Sampling reported	LR
Larrat	Consecutive recruitment, but of known cases and known negative controls	HR	blinded	LR	Blinded	LR	Sampling reported, same reference standard	LR
Lee	Consecutive recruitment	LR	Not blinded, interpretation unbiased	LR	Not blinded, interpretation unbiased	LR	Sampling reported, same reference standard	LR
Lukacs	NR	UR	NR	UR	NR	UR	Sampling reported, same reference standard	LR
McCarron	Case control, known positive and negative cases from prevalence survey	HR	NR	UR	NR	UR	NR	UR
Marques 2012	No case control design	LR	Not blinded, interpretation unbiased	LR	Not blinded, interpretation unbiased	LR	Sampling reported, same reference standard	LR
Marques 2016	NR	UR	Not blinded, interpretation unbiased	UR	Not blinded, interpretation unbiased	UR	NR, same reference standard, NR	UR
Mossner	Sampling from high-risk and low risk groups	UR	Not blinded, interpretation unbiased	UR	Not blinded, interpretation unbiased	UR	Sampling reported, same reference standard, all patients included in analysis	LR
Nandagopal	NR	UR	NR	UR	NR	UR	NR	UR
O Brien	No case control design,	LR	Blinded	LR	Blinded	LR	Sampling partly reported, same reference standard	LR
Parker	Case control design	HR	Not blinded, interpretation unbiased	LR	Not blinded, interpretation unbiased	LR	Sampling partly reported, same reference standard	LR
Ross	Possible case control design, sampling NR	HR	Not blinded, interpretation unbiased	LR	Not blinded, interpretation unbiased	LR	Flow reported	LR
Sheperd	No case control design, but partly sampling from patients with known disease	LR	Not blinded, interpretation unbiased	LR	Not blinded, interpretation unbiased	LR	NR	UR
Soulier	Sampling from high-risk and low risk-groups	HR	Not blinded, interpretation unbiased	LR	Not blinded, interpretation unbiased	LR	NR, same reference standard, NR	LR
Tejada-Strop	Case control	HR	Not blinded, interpretation unbiased	LR	Not blinded, interpretation unbiased	LR	NR	UR
Tuaillon, E	Case control	HR	Blinded	LR	Blinded	LR	Sampling reported, same reference standard	LR
Waterboer, T	No case control	LR	Not blinded, interpretation unbiased	LR	Not blinded, interpretation unbiased	LR	NR	UR

Abbreviations: LR: low risk, HR: high risk, UR: unknown risk, NR: not reported; shaded: low risk of bias

#### Diagnostic performance

**Fig. 3:**
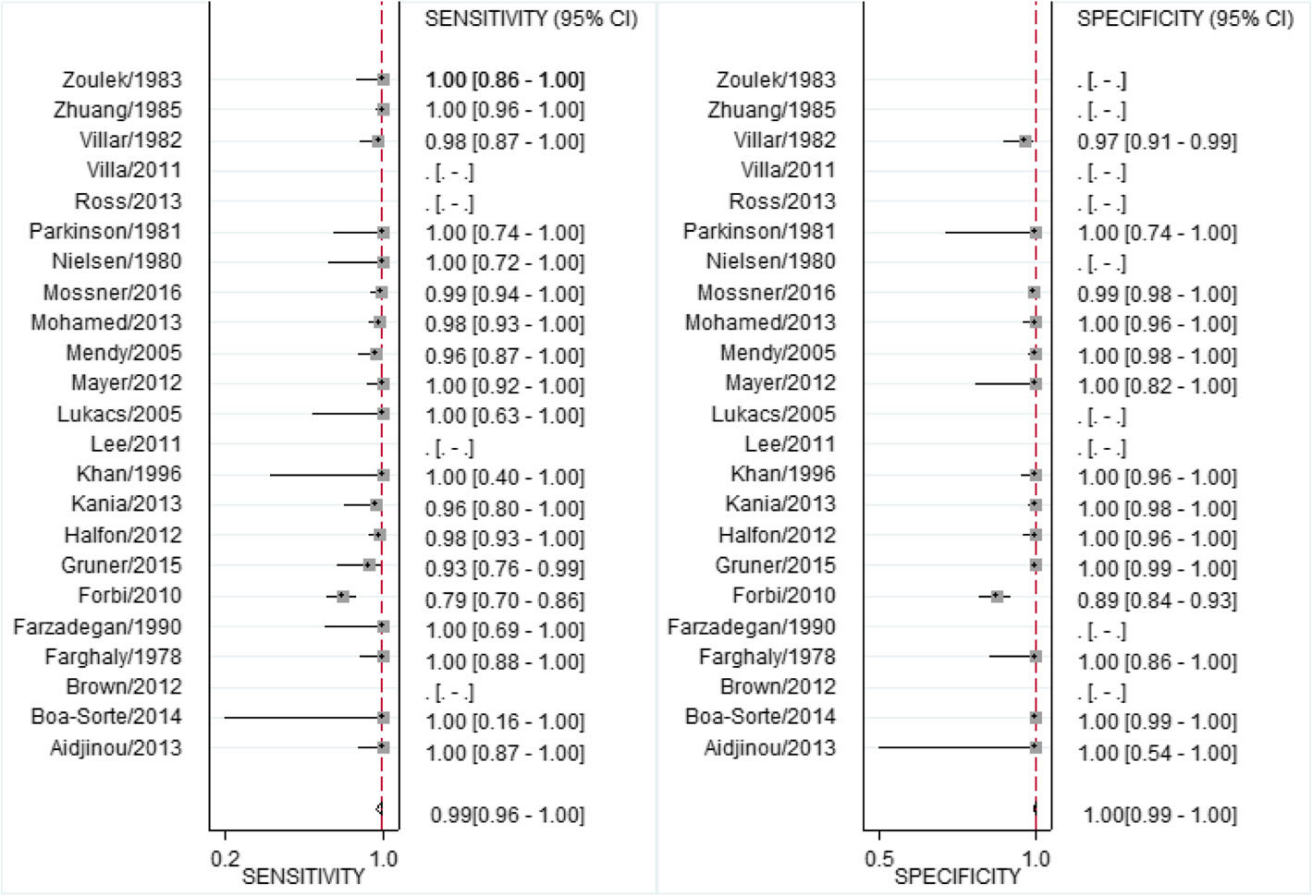
Forest plot of sensitivity and specificity of hepatitis B Surface antigen serological diagnosis in DBS compared to venous blood samples

In general, based on the 19 studies evaluated, testing **for HBsAg** using DBS maintained good accuracy compared to testing using plasma or serum from venipuncture. Reported sensitivity estimates ranged from 79%–100% with a pooled estimate from a meta-analysis of 98% (95%CI 95%–99%), and specificity ranged from 89%–100%, with a pooled estimate of 100% (95%CI 99–100%) (Fig. 3). The positive likelihood ratio was 703 (95%CI 107–4615) and the negative likelihood ratio was 0.02 (95% CI 0.008–0.04). Bivariate and univariate estimates of pooled sensitivity and specificity were similar.

**Fig. 4:**
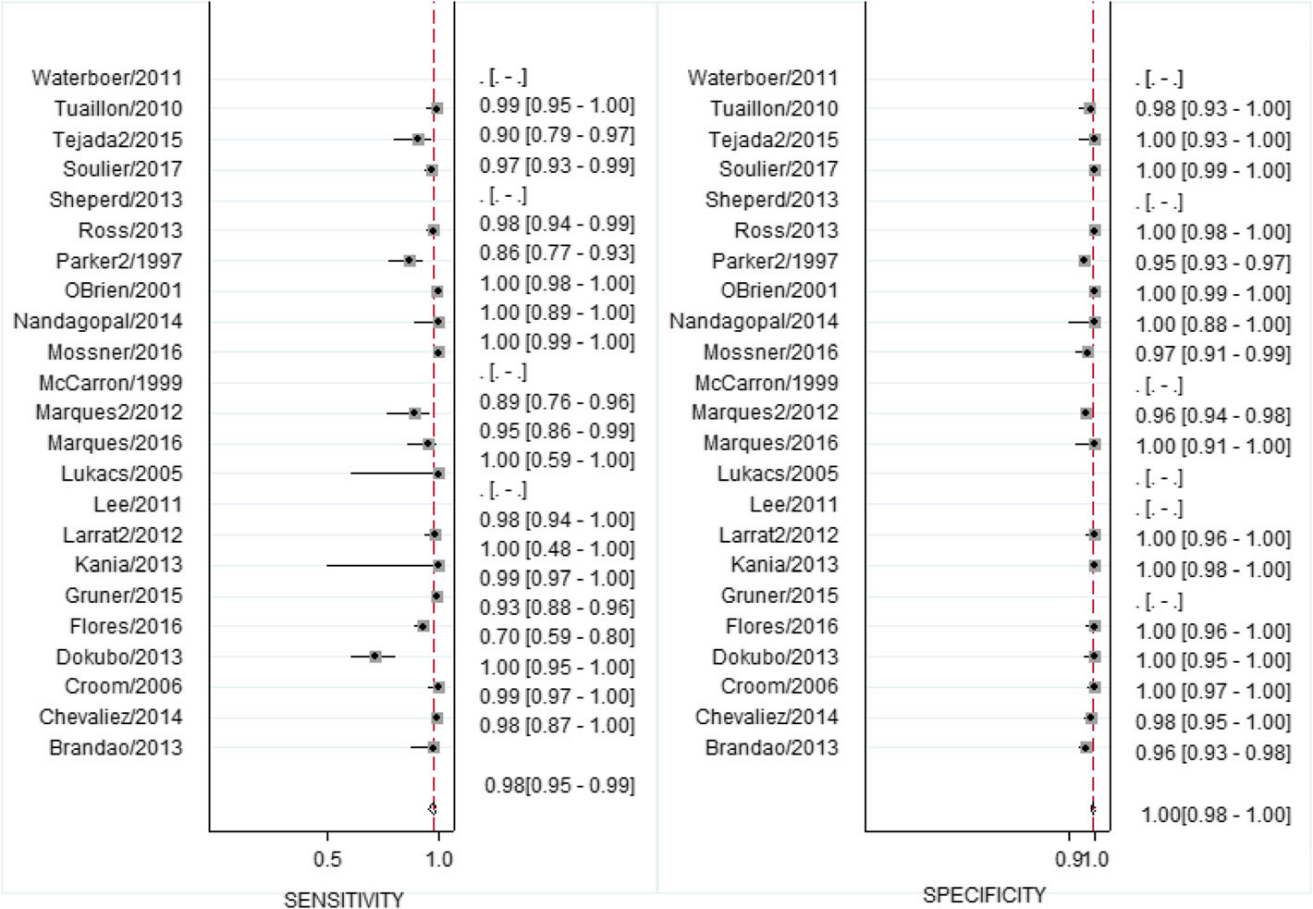
Forest plot of sensitivity and specificity of hepatitis C antibody detection in DBS samples compared to venous blood samples

Based on the 19 studies included in the quantitative meta-analysis **for HCV antibody**, the reported sensitivity for HCV-Ab using DBS ranged from 70% to 100% and specificity ranged from 95 to 100%. The pooled bivariate estimate of sensitivity and specificity was 98% (95%CI 95–99) and 99% (95%CI 98–100), respectively (Fig. 4). The positive likelihood ratio was 361 (95%CI 61–2163) and the negative likelihood ratio 0.02 (95%CI 0.01–0.05). Four included studies reported also measures of agreement with kappa values ranging from 0.87–1 between DBS and venous blood samples [26, 39, 59, 60]. Bivariate and univariate estimates of pooled sensitivity and specificity were similar.

Visual assessment as well τ^2^ and its *p*-value showed moderate heterogeneity in the bivariate analysis of studies. To further assess the heterogeneity, we evaluated different parameters with potential to affect accuracy, including assay type and cut-off used, and storage conditions.

##### Effect of test and cut-off used

There are no standardised cut-offs for **HBsAg** detection using DBS. Eight of the studies reported a cut-off based on ROC analysis from the same set of samples as the validation set. Several of the included studies noted that the ideal cut-off (as suggested by ROC curves) for determining test positivity should be higher for DBS samples than for plasma or serum samples. Other studies have indicated that this was due to the small sample volume used in DBS (commonly 50 μl). Indeed the one study with low sensitivity (79%) and specificity (89%) [36] only used 25 μl of blood on filter paper (37).

Different assays were used in **HCV-Ab detection**. Cut-offs varied widely and as no standardized cut-offs exist, investigators for many studies determined their own cut-off via ROC curves using the same set of samples. Nine of the included studies reported on cut-offs used for DBS [26, 27, 40, 59, 60, 64–66]. Stratification by type of test or cut-off used was not possible as the number of strata would have been large and the results difficult to interpret. Stratification in a pooled analysis based on amount of blood (50 μl versus >50 μl) or sampling method (venous blood vs capillary blood) showed similar sensitivity and specificity estimates (data not shown).

##### Effect of storage conditions

For **HBsAg detection,** the effect of a range of storage conditions was evaluated in six studies, including storage temperature ranging from −20 to 33 °C and storage duration ranging from overnight to 180 days. In general, storage at room temperature or higher (30–33 °C) did not clearly affect accuracy of testing and no decrease in sensitivity was found with prolonged storage at ambient temperatures [34, 44–46, 48, 51, 54].

Four studies of diagnostic accuracy for **HCV-Ab** using DBS samples evaluated different storage conditions in a subset of samples that did not contribute to the diagnostic accuracy evaluations. In one study, three out of three previously negative samples exceeded cut-off values (i.e. would have been interpreted as positive) after storage for 3 days at room temperature [66]. Similarly, one of the included studies showed that after 6 days of storage at room temperature, the cut-off values were exceeded and previously negative samples became positive [63]. Two studies showed relative stability at room temperature for up to 60 days, but found that storage at −20 °C was associated with less variation in quantitative values [26, 59].

One of the 19 studies contributing to the diagnostic accuracy calculations kept study samples at room temperature for more than 24 h [55]. A pooled analysis stratifying studies according to whether samples had been left at room temperature for longer than 4 h or not did not find any difference in performance and so did not explain the heterogeneity observed in the meta-analysis (data not shown).

##### Sensitivity analysis

In a sensitivity analysis, we found no difference in estimates of diagnostic performance between those studies that reported consecutive sampling and those with case-control study design (data not shown).

## Discussion

To our knowledge, this is the first comprehensive and systematic review to summarize the utility of DBS for HCV-Ab and HBsAg testing. Overall, we found very good diagnostic accuracy and precision for detection of HCV-Ab and HBsAg using DBS samples. These findings were based on a larger number of studies compared to the companion systematic reviews undertaken for HBV DNA and HCV RNA using DBS [30] and were rated as moderate quality. This provides support for the use of DBS from capillary blood for diagnostic testing of HBV or HCV, especially in community settings and hard to reach populations where there is limited access to venipuncture or inadequate laboratory infrastructure to prepare or transport plasma samples or limited access to rapid diagnostic tests.

The WHO 2017 testing guidelines recommended the use of a single quality-assured serological assay (i.e either a laboratory-based EIA or RDT to detect HBsAg and anti-HCV that meet minimum performance standards, and where possible delivered at the point of care to improve access and linkage to care and treatment. The guidelines also made a conditional recommendation to consider the option of DBS specimens for HBsAg and HCV-Ab serology testings in settings where there are no facilities or expertise to take venous whole blood specimens; or RDTs are not available or their use is not feasible, or there are persons with poor venous access (e.g. drug treatment programmes, prisons) [29]. This recommendation was rated as conditional mainly because of the slightly lower accuracy using DBS compared to venous blood sampling and uncertainty regarding the generalizability of studies included in this review (in particular regarding impact of different storage and transport conditions).

There were several limitations to this review. First, we did not include studies in languages other than English and no unpublished data from laboratories were included. Second, while only a few studies were rated as having a low risk of bias, overall the quality of evidence from studies was rated as moderate. Third, most studies did not use fresh samples and no uniform protocol was applied. Fourth, there is also a risk of overestimation of pooled sensitivity and specificity by including studies that applied cut-off levels derived from the same study population. No stratified analysis was possible for the type of test within this review. Therefore, we are unable to recommend the use of certain commercial tests over others using DBS testing of HCV-Ab or HBsAg or to suggest a cut-off specific to a test that should be used for DBS samples.

Several studies found that when assessing stability in separate sample sets samples became false-positive after longer exposure at ambient temperatures for both HCV-Ab [27, 66] and HBsAg [44, 45]. Further data is needed to understand the stability of DBS with different environmental conditions (i.e. temperature and humidity). The review highlights the need for standardized validation of specific tests with DBS.

While some studies published detailed protocols on how to collect and analyze DBS [37], no manufacturers to date have provided instructions on how to use their assays with DBS (including processing methods and possibly different cut-offs). There is a need for manufacturers to validate their assays and provide instructions for the use of DBS even if no claim for regulatory approval is made.

### Implementation of DBS and future work:

Consideration of the use DBS sampling for HBV and C serological or nucleic acid testing or both will depend on the health-care setting and infrastructure, and epidemiological context. If good-quality RDTs are available that can be performed using capillary blood then the focus may be more on prioritizing DBS for NAT testing of HBV DNA and HCV RNA. However, if RDTs are not available and there are no facilities or expertise to take venous blood samples, then DBS testing may be equally important to increase access to serological testing as well as NAT. Conveniently, both could be performed from the same specimen. A further situation where DBS may be applicable is where large numbers of individuals are being tested simultaneously. DBS may also be useful where polyvalent screening for multiple diseases is done, but where multiplex RDTs for this purpose are not available or are more costly. The adoption of DBS sampling in a hepatitis testing programme requires the availability of a centralized laboratory competent at handling and processing this sample type. The current lack of validation of assays on this sample type for HBsAg or HCV-Ab and manufacturer’s guidance on the optimal pre-analytical treatment of specimens makes quality control challenging.

Further data is also needed on demonstrating the stability of DBS with individual tests under different field transport and storage conditions likely to be encountered in low resource settings. as well as on the type of test used and how long DBS samples can be left at different temperature or humidity levels. Since DBS requires only a small sample of blood to maintain sensitivity, a higher analytical cut-off may be required to maintain sensitivity and overcome variability at the lower end of the dynamic range of the test as compared to higher volume plasma samples – even if in our limited stratified analysis on this we did not find an effect of blood volume on diagnostic accuracy of HBV or HCV. Further insight can also be gained from individual patient analyses of the existing data, which was beyond the scope of this review.

## Conclusion

While diagnostic accuracy of DBS for HCV-Ab and HBsAg testing is adequate in studies included in this review, lack of standardization of testing protocols and uncertainty about their use in field conditions and the appropriate assay cut-offs limits the wider application of DBS.
